# Correction: Precise excitation-inhibition balance controls gain and timing in the hippocampus

**DOI:** 10.7554/eLife.71338

**Published:** 2021-06-24

**Authors:** Aanchal Bhatia, Sahil Moza, Upinder Singh Bhalla

Bhatia A, Moza S, Bhalla US. 2019. Precise excitation-inhibition balance controls gain and timing in the hippocampus. *eLife* **8**:e43415. doi: 10.7554/eLife.43415.

Published 25, April 2019

#1 Figure 2 supplement figures 1 and 2 were swapped.

The error arose due to an oversight when we compiled the manuscript. The legends are consistent with the references in text, but the figure panels had been swapped.

#2 and #3: We inadvertently mentioned the concentrations of 3 chemicals from an older protocol that we had used. High sucrose artificial cerebro-spinal fluid (hsaCSF) did not contain glucose, NaH_2_PO_4_ in aCSF was 1.25mM and we used Na_4_-EGTA in the internal solution (not EGTA). All other chemicals and concentrations remain unchanged. The revised concentrations from the current protocol are underlined in the corrected text below.

#2 Methods (Slice preparation)

Original text:

Slices (350 microns) were cut in ice-cold high sucrose ASCF containing (in mM): 87 NaCl, 2.5 KCl, 1.25 NaH_2_PO_4_, 25 NaHCO_3_, 75 sucrose, 10 glucose, 0.5 CaCl_2_, 7 MgCl_2_. Slices were stored in a holding chamber, in artificial cerebro-spinal fluid (aCSF) containing (in mM) - 124 NaCl, 2.7 KCl, 2 CaCl_2_, 1.3 MgCl_2_, 0.4 NaH_2_PO_4_, 26 NaHCO_3_, and 10 glucose, saturated with 95% O_2_/5% CO_2_.

Corrected text:

Slices (350 microns) were cut in ice-cold high sucrose artificial cerebro-spinal fluid (hsaCSF) containing (in mM): 87 NaCl, 2.5 KCl, 1.25 NaH_2_PO_4_, 25 NaHCO_3_, 75 sucrose, 0.5 CaCl_2_, 7 MgCl_2_. Slices were stored in a holding chamber, in artificial cerebro-spinal fluid (aCSF) containing (in mM) - 124 NaCl, 2.7 KCl, 2 CaCl_2_, 1.3 MgCl_2_, 1.25 NaH_2_PO_4_, 26 NaHCO_3_, and 10 glucose, saturated with 95% O_2_/5% CO_2_.

#3 Methods (Electrophysiology)

Original text:

Pipettes were filled with internal solution containing (in mM): 130 K-gluconate, 5 NaCl, 10 HEPES, 1 EGTA, 2 MgCl_2_, 2 Mg-ATP, 0.5 Na-GTP and 10 Phosphocreatinine, pH adjusted to 7.3, osmolarity ~285 mOsm.

Corrected text:

Pipettes were filled with internal solution containing (in mM): 130 K-gluconate, 5 NaCl, 10 HEPES, 1 Na_4_-EGTA, 2 MgCl_2_, 2 Mg-ATP, 0.5 Na-GTP and 10 Phosphocreatinine, pH adjusted to 7.3, osmolarity ~285 mOsm.

#4

Additionally, owing to a production error the equal contributions for first and second author Aanchal Bhatia and Sahil Moza was erroneously omitted from the published version.

The article has been corrected accordingly.

