# Supplementary material for: Precise excitation-inhibition balance controls gain and timing in the hippocampus
Source: eLife. 2019 Apr 25;8:e43415. doi: 10.7554/eLife.43415 (PMC6517031; doi:10.7554/eLife.43415)
Supplement: Supplementary file 1. — The median, 25% and 75% values for each of the four distributions in Figure 6—figure supplement 1e are shown. [file elife-43415-supp1.docx]

| **Name (Units)** | **Median** | **25%** | **75%** |
| --- | --- | --- | --- |
| Excitatory $\tau_{\mathrm{rise}}$ (ms) | 0.007 | 0.005 | 0.011 |
| Excitatory $\tau_{\mathrm{decay}}$ (ms) | 0.016 | 0.011 | 0.020 |
| Inhibitory $\tau_{\mathrm{rise}}$ (ms) | 0.013 | 0.005 | 0.020 |
| Inhibitory $\tau_{\mathrm{decay}}$ (ms) | 0.027 | 0.020 | 0.038 |
