# Supplementary material for: Precise excitation-inhibition balance controls gain and timing in the hippocampus
Source: eLife. 2019 Apr 25;8:e43415. doi: 10.7554/eLife.43415 (PMC6517031; doi:10.7554/eLife.43415)
Supplement: Supplementary file 2. — Parameters for this model were either calculated using electrophysiological experimental conditions, taken from literature (Table S2a) or fit from data (Table S2b). [file elife-43415-supp2.docx]

**S2a. Values taken from literature**

| **Variable** | **Meaning** | **Source** | **Value** |
| --- | --- | --- | --- |
| $g_{\mathrm{leak}}$ | Leak conductance | Fernandos and White, J. Neuro. (2010) | 10 nS |
| $E_{\mathrm{exc}}$ | Excitatory reversal | Calculated (**Methods**) | 0 mV |
| $E_{\mathrm{inh}}$ | Inhibitory reversal | Calculated (**Methods**) | -70 mV |
| $E_{\mathrm{leak}}$ | Leak reversal | Fernandos and White, J. Neuro. (2010) | -65 mV |
| $C_{m}$ | Membrane capacitance | neuroelectro.org | 100 pF |

**S2b. Values extracted by fitting data**

| **Variable** | **Meaning** | **Range (units)** |
| --- | --- | --- |
| $t$ | Time | 0-100 ms |
| $g_{\mathrm{exc}}$ | Excitatory max conductance | 0 - 5 nS |
| $\tau_{\mathrm{exc}}^{\mathrm{rise}}$ | Excitatory Rise | 7 ms |
| $\tau_{\mathrm{exc}}^{\mathrm{decay}}$ | Excitatory Fall | 16 ms |
| $\delta_{\mathrm{exc}}^{\mathrm{onset}}$ | Excitatory onset time | 0 ms |
| $P$ | I/E ratio | 0 - 5 |
| $g_{\mathrm{inh}}$ | Inhibitory max conductance | $P$ x $g_{\mathrm{exc}}$ |
| $\tau_{\mathrm{inh}}^{\mathrm{rise}}$ | Inhibitory Rise | 13 ms |
| $\tau_{\mathrm{inh}}^{\mathrm{decay}}$ | Inhibitory Fall | 27 ms |
| $\delta_{\mathrm{inh}}^{\mathrm{onset}}$ | Inhibitory onset time | 2-15 ms |
