# Supplementary material for: Precise excitation-inhibition balance controls gain and timing in the hippocampus
Source: eLife. 2019 Apr 25;8:e43415. doi: 10.7554/eLife.43415 (PMC6517031; doi:10.7554/eLife.43415)
Supplement: Supplementary file 3. — Parameters for this model were either calculated using electrophysiological experimental conditions, or taken from literature. The simulations were conducted using synaptic conductances, measured from voltage clamp data (Figure 2). [file elife-43415-supp3.docx]

| **Variable** | **Meaning** | **Value** |
| --- | --- | --- |
| $l$ | Soma length (cylindrical compartment) | 10 μ |
| $d$ | Soma diameter | 10 μ |
| $\tau_{m}$ | Membrane time constant | 0.01 s |
| $E_{\mathrm{exc}}$ | Excitatory reversal | 0 mV |
| $E_{\mathrm{inh}}$ | Inhibitory reversal | -70 mV |
| $E_{\mathrm{leak}}$ | Leak reversal | -65 mV |
| $E_{\mathrm{Ns}}$ | Sodium channel reversal potential | 55 mV |
| $E_{\mathrm{KDR}}$ | Delayed rectifier potassium channel reversal potential | -75 mV |
| $g_{\mathrm{Ns}}$ | Sodium channel maximum conductance | 20 mS/cm^2^ |
| $g_{\mathrm{KDR}}$ | Delayed rectifier potassium channel maximum conductance | 25 mS/cm^2^ |
